# ENT3: A lysosomal urate transporter regulating urate disposition and macrophage inflammation

**DOI:** 10.1016/j.isci.2025.114249

**Published:** 2025-11-27

**Authors:** Isamu Matake, Tomoya Yasujima, Hirotaka Matsuo, Akiyoshi Nakayama, Yu Toyoda, Tappei Takada, Katsuhisa Inoue, Takahiro Yamashiro, Hiroaki Yuasa

**Affiliations:** 1 Department of Biopharmaceutics, Graduate School of Pharmaceutical Sciences, Nagoya City University, Nagoya, Aichi, Japan; 2 Department of Integrative Physiology and Bio-Nano Medicine, National Defense Medical College, Tokorozawa, Saitama, Japan; 3 Department of Bioinformation Management, National Defense Medical College Research Institute, Tokorozawa, Saitama, Japan; 4 Department of Pharmacy, The University of Tokyo Hospital, Tokyo, Japan; 5 Department of Biopharmaceutics, School of Pharmacy, Tokyo University of Pharmacy and Life Sciences, Hachioji, Tokyo, Japan

**Keywords:** Biochemistry, Immunology, Cell biology

## Abstract

Urate is the final oxidation product of purine metabolism in humans, and its extracellular accumulation leads to the formation of monosodium urate (MSU) crystals that trigger gout. Although several plasma membrane transporters involved in urate reabsorption and excretion have been identified, the mechanisms governing intracellular urate clearance remain unclear. Here, we show that equilibrative nucleoside transporter 3 (ENT3/SLC29A3) functions as a proton-coupled urate exporter from lysosomes. Using a recombinant ENT3 that localizes to the plasma membrane, we determined its urate transport kinetics (*K*_m_ ≈ 1.15 mM), establishing ENT3 as a low-affinity, high-capacity urate transporter. In differentiated THP-1 macrophage-like cells, ENT3 knockdown impaired clearance of phagocytosed MSU, while reducing interleukin-1β secretion likely due to diminished adenosine-mediated inflammatory signaling. These findings reveal an unrecognized role of ENT3 in lysosomal urate handling and inflammation, suggesting that ENT3 dysfunction may contribute to gout and other urate-associated disorders.

## Highlights

• ENT3 is a proton-coupled lysosomal urate exporter with low affinity and high capacity
• Disease-associated ENT3 mutations abolish urate transport
• ENT3 knockdown in macrophage-like cells impairs lysosomal urate clearance
• ENT3 regulates inflammasome activation via dual roles in urate and adenosine transport

## Graphical abstract

**Figure:**
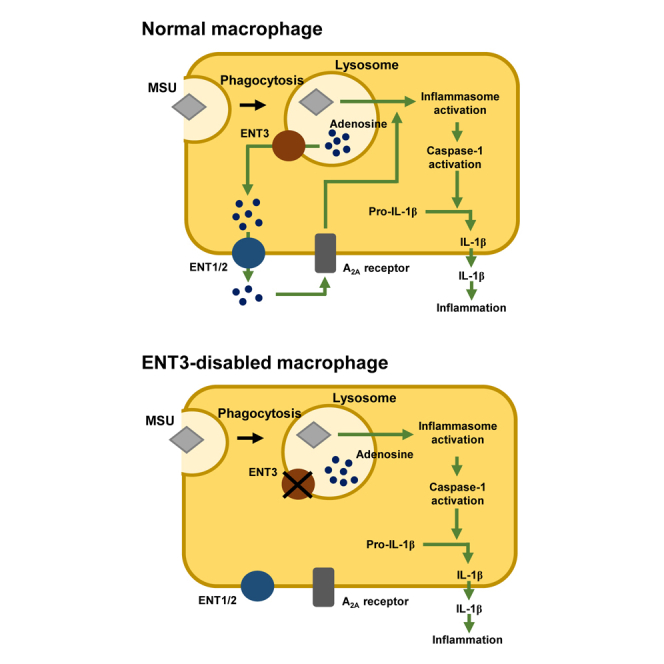

## Introduction

Gout is a common form of inflammatory arthritis that develops when serum urate concentrations exceed the physiological solubility threshold, leading to the formation of monosodium urate (MSU) crystals.^1^^,^^2^ Under normal conditions, urate remains soluble in blood; however, when its concentration exceeds ∼7 mg/dL (∼400 μM)—the saturation point—crystallization can occur.^3^ These MSU crystals circulate and preferentially deposit in joint tissues, where they serve as potent activators of the innate immune system and trigger acute gout flares.^4^

Once deposited in joint tissues, MSU crystals are readily recognized and internalized by macrophages, which act as key effector cells in the inflammatory cascade.^4^^,^^5^ Following phagocytosis, the crystals are trafficked to lysosomes, specialized organelles responsible for degrading and recycling cellular components. However, MSU crystals are structurally resistant to lysosomal degradation and can compromise lysosomal integrity by physically disrupting the membrane.^6^ This disruption activates the NOD-like receptor family pyrin domain containing 3 (NLRP3) inflammasome, a cytosolic multiprotein complex that drives the maturation and secretion of pro-inflammatory cytokines, particularly interleukin-1β (IL-1β).^4^^,^^7^ IL-1β subsequently orchestrates the inflammatory response underlying the clinical manifestations of gout, including intense joint pain, erythema, and swelling.^8^^,^^9^

Although hyperuricemia is a well-established risk factor for gout, not all individuals with elevated serum urate levels develop the disease.^10^^,^^11^ Epidemiological data indicate elevation of gout risk with increasing serum urate level; for instance, the 5-year incidence of gout is approximately 4.1% in individuals with urate levels between 8.0 and 8.9 mg/dL, 19.8% at 9.0–9.9 mg/dL, and 30% at levels exceeding 10 mg/dL.^12^ Nevertheless, many individuals with serum urate concentrations well above the solubility threshold remain asymptomatic. These observations suggest that urate supersaturation and extracellular MSU crystallization alone are insufficient to trigger gout, implicating additional cellular mechanisms in the regulation of crystal formation and inflammatory activation.

A plausible hypothesis is that efficient intracellular urate clearance mechanisms, particularly within macrophages, serve as protective factors by limiting MSU accumulation and preventing inflammasome activation. Specifically, if intralysosomal urate concentrations remain below the saturation threshold, even internalized MSU crystals may dissolve over time, thereby averting sustained lysosomal damage. This process would require carrier-mediated transport systems capable of exporting urate from the lysosomal lumen to the cytosol for subsequent excretion.

Recently, we identified equilibrative nucleoside transporter 2 (ENT2/SLC29A2), a plasma membrane-localized member of the SLC29 family known for transporting nucleosides via facilitated diffusion, as a novel urate transporter.^13^ Building on this finding, we hypothesized that ENT3/SLC29A3, a lysosomal membrane-localized member of the same family,^14^ might similarly participate in urate transport within intracellular compartments, particularly in lysosomes. Although ENT3 is traditionally recognized for mediating nucleoside transport in a proton-dependent manner, its involvement in urate transport has not been explored yet. In the present study, we demonstrate for the first time that ENT3 exhibits urate transport activity and functions as a proton-dependent urate exporter from lysosomes. Additionally, we also delineate the impact of the functionality of ENT3 on inflammatory responses, in which concurrent ENT3-mediated adenosine efflux may play a role for adenosine-dependent enhancement,^15^ oppositely to the putative role of ENT3-mediated urate efflux for suppression, in differentiated THP-1 cells as a macrophage-like model. Altogether, the present study provides novel insights into the potential role of ENT3 in urate disposition and inflammatory responses in macrophages.

## Results and discussion

### Identification of urate transport activity by ENT3

**Figure 1:**
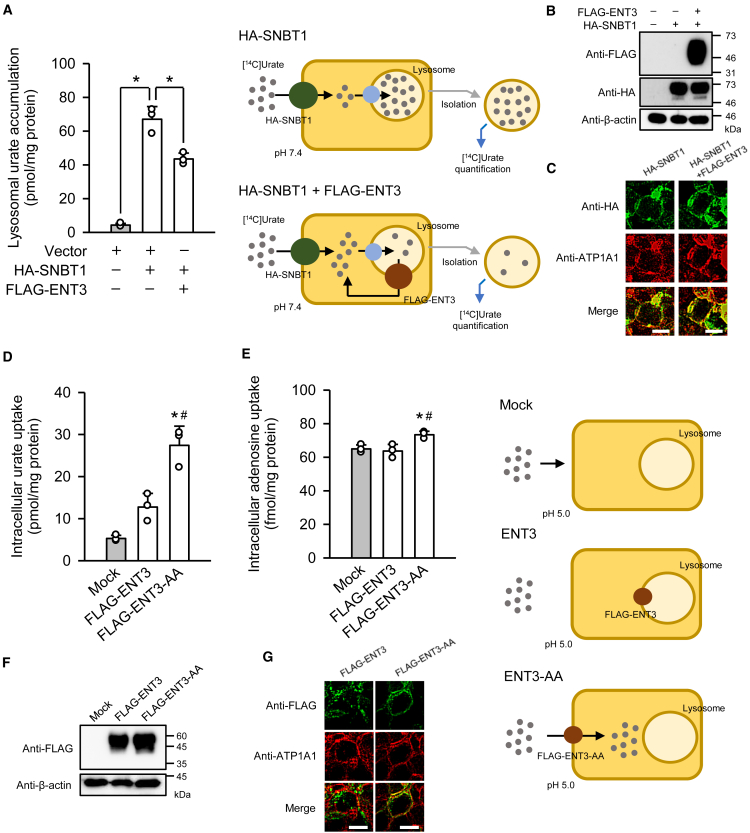
Urate transport activity of ENT3 and ENT3-AA (A) Lysosomal urate accumulation was assessed by isolating lysosomes after cellular uptake of [^14^C]urate (10 μM) for 30 min at pH 7.4 and 37°C in HEK293 cells transiently transfected with the plasmid encoding HA-SNBT1 (0.5 μg), with or without co-transfection of FLAG-ENT3 plasmid (0.5 μg). (B) Western blotting was performed using anti-FLAG and anti-HA antibodies on whole-cell lysates (10 μg protein per lane) prepared from HEK293 cells transiently expressing HA-SNBT1, with or without FLAG-ENT3. β-actin blots are shown as loading controls. (C) Immunofluorescence analysis revealed co-localization of HA-SNBT1 (green) with ATP1A1 (red), a plasma membrane marker, in transiently transfected HEK293 cells. Scale bars: 10 μm (D) Uptake of [^14^C]urate (4 μM) was measured over 2 min at pH 5.0 and 37°C in HEK293 cells transiently expressing FLAG-ENT3 or FLAG-ENT3-AA, or mock cells. (E) Uptake of [^3^H]adenosine (1 nM) was similarly assessed over 2 min at pH 5.0 and 37°C in the same cell groups. (F) Western blotting was performed using anti-FLAG antibody on whole-cell lysates (10 μg protein per lane) prepared from HEK293 cells transiently expressing FLAG-ENT3 or FLAG-ENT3-AA, or mock cells. β-actin blots are shown as loading controls. (G) Immunofluorescence images show the localization of FLAG-ENT3 and FLAG-ENT3-AA (green) in relation to ATP1A1 (red) in transiently transfected HEK293 cells. Scale bars: 10 μm. Data are presented as the mean ± SD from three biological replicates using separate cell preparations. Statistical significance was evaluated using two-way (A) or one-way (D and E) ANOVA followed by Bonferroni post-hoc test. ^∗^*p* < 0.05 for the indicated comparison (A); ^∗^*p* < 0.05 versus mock cells (D and E), ^#^*p* < 0.05 versus FLAG-ENT3-expressing cells (D and E).

To determine whether ENT3 functions as a urate transporter at the lysosomal membrane, we assessed lysosomal urate accumulation in HEK293 cells transiently transfected with rat sodium-dependent nucleobase transporter 1 (SNBT1/SLC23A4) tagged with HA (HA-SNBT1) and human ENT3 tagged with FLAG (FLAG-ENT3), as shown in Figure 1A. Cells were incubated with [^14^C]urate (10 μM) for 30 min at pH 7.4, followed by lysosome isolation and quantification of accumulated radioactivity. In cells expressing HA-SNBT1 alone, a marked increase in lysosomal [^14^C]urate accumulation was observed. This finding is consistent with the known ability of HA-SNBT1 to facilitate concentrative urate uptake into the cytosol via Na^+^ co-transport,^16^ followed by subsequent putative carrier-mediated transfer into lysosomes. Notably, co-expression of FLAG-ENT3 significantly reduced lysosomal [^14^C]urate accumulation, suggesting that ENT3 facilitates urate efflux from lysosomes.

To confirm that this effect was not due to altered expression or localization of HA-SNBT1, western blot analysis was performed. The analysis indicated that the HA-SNBT1 protein level remained unchanged in the presence of FLAG-ENT3 (Figure 1B), excluding the possibility of post-translational downregulation or degradation. Moreover, in immunofluorescence analysis (Figure 1C), HA-SNBT1 was colocalized with ATP1A1, a plasma membrane marker, under both single expression and co-expression conditions, indicating that FLAG-ENT3 co-expression did not affect HA-SNBT1 localization.

To directly evaluate the transport activity of ENT3 in a controlled system, we generated a recombinant ENT3 protein (ENT3-AA), in which the dileucines (L^31^L^32^) in the N-terminal lysosomal targeting motif (D^27^QEAL^31^L^32^) was replaced with dialanines (A^31^A^32^) to thereby redirect the protein to the plasma membrane.^14^ The use of ENT3-AA, which was tagged with FLAG in this set of experiments, enabled direct uptake assays using extracellular radiolabeled substrates. When assessed under an acidic condition (pH 5.0), which mimics the lysosomal environment, in transiently transfected HEK293 cells (Figure 1D), FLAG-ENT3-AA-transfected cells exhibited significantly increased uptake of [^14^C]urate (4 μM) compared with mock-transfected cells, whereas FLAG-ENT3-transfected cells did not. These results indicate that plasma membrane-localized FLAG-ENT3-AA could operate for cellular urate uptake, whereas lysosomal membrane-localized FLAG-ENT3 could not exhibit activity for that. Consistently, FLAG-ENT3-AA retained its nucleoside transport capability at the plasma membrane, as evidenced by the increased uptake of [^3^H]adenosine (1 nM) in FLAG-ENT3-AA-transfected cells relative to mock-transfected cells (Figure 1E), indicating that redirection to the plasma membrane did not compromise substrate specificity or transport function. In FLAG-ENT3-transfected cells, [^3^H]adenosine uptake was confirmed not to be increased.

Western blot analysis confirmed the expression of FLAG-ENT3-AA (Figure 1F), and immunofluorescence analysis demonstrated successful redirection of the protein to the plasma membrane (Figure 1G). FLAG-ENT3 was confirmed to be expressed at a comparable level and localized intracellularly. These results validate the ENT3-AA construct as a suitable model for functional and kinetic analyses of urate transport.

Collectively, these findings establish that ENT3 exhibits urate transport activity under acidic conditions and likely serves as a lysosomal urate exporter that operates via a proton-dependent mechanism, as hypothesized. This study provides the first direct evidence implicating ENT3 in subcellular urate handling and offers mechanistic insight into the regulation of intracellular urate homeostasis.

### Kinetic analysis of ENT3-AA-mediated urate transport

**Figure 2:**
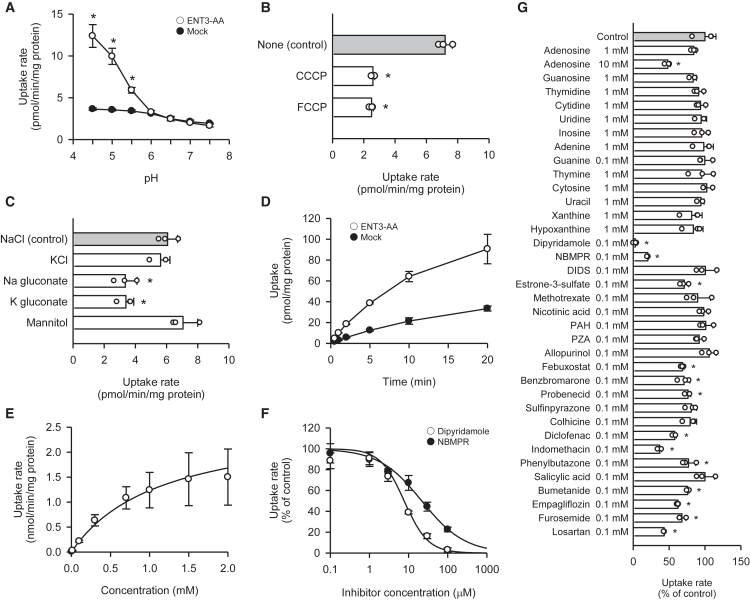
Functional characteristics of ENT3-AA in urate transport in HEK293 cells transiently expressing ENT3-AA (A) The effect of pH on [^14^C]urate (4 μM) uptake was evaluated over 2 min at 37°C. (B) The effect of protonophores on ENT3-AA-mediated [^14^C]urate (4 μM) uptake was assessed over 2 min at pH 5.0 and 37°C in the presence or absence of a protonophore (50 μM). CCCP, carbonyl cyanide *m*-chlorophenylhydrazone; FCCP, carbonyl cyanide *p*-trifluoromethoxyphenylhydrazone. (C) The effect of ionic composition on ENT3-AA-mediated [^14^C]urate (4 μM) uptake was evaluated over 2 min at pH 5.0 and 37°C by substituting NaCl with the indicated salts or mannitol. (D) Time-course of [^14^C]urate (4 μM) uptake was evaluated at pH 5.0 and 37°C. (E) Concentration-dependent uptake of [^14^C]urate by ENT3-AA was measured over 2 min at pH 5.0 and 37°C. The estimated *V*_max_ and *K*_m_ values were 2.65 ± 1.28 nmol/min/mg protein and 1.15 ± 0.29 mM, respectively. (F) Inhibitory effects of specific ENT inhibitors on ENT3-AA-mediated [^14^C]urate (4 μM) uptake were evaluated over 2 min at pH 5.0 and 37°C. The *IC*_50_ values for dipyridamole and nitrobenzylthioinosine (NBMPR) were 7.71 ± 0.80 μM and 21.7 ± 5.5 μM, respectively, and the *n* values were 1.43 ± 0.40 and 0.793 ± 0.128, respectively. (G) Effect of various compounds on ENT3-AA-mediated [^14^C]urate (4 μM) uptake was evaluated over 2 min at pH 5.0 and 37°C. DIDS, 4,4′-diisothiocyano-2,2′-stilbenedisulfonic acid; PAH, *p*-aminohippurate; PZA, pyrazine carboxylic acid. Data are presented as the mean ± SD from three biological replicates using separate cell preparations. Statistical significance was evaluated using Student’s *t* test (A) and one-way ANOVA followed by Dunnett’s test (B, C, and G). ^∗^*p* < 0.05 versus mock cells at each pH (A) or versus control (B, C, and G).

To characterize the transport properties of ENT3, we first examined the pH dependence of urate uptake mediated by the ENT3-AA construct in transiently transfected HEK293 cells. As shown in Figure 2A, the uptake of [^14^C]urate (4 μM) in ENT3-AA-transfected cells was maximal at pH 4.5 and declined progressively with increasing extracellular pH, reaching the level comparable to the consistently low level in mock-transfected cells at pH ≥ 6.0. This pH-dependent profile indicates that ENT3-AA functions optimally under acidic conditions, requiring proton for urate transport, and also that the condition of pH 5.0 in regular uptake assays was suitable to gain sufficiently high urate transport activity for functional analyses. To determine whether urate transport by ENT3-AA is driven by the proton gradient, we evaluated the effect of the protonophores, carbonyl cyanide *m*-chlorophenylhydrazone and carbonyl cyanide *p*-trifluoromethoxyphenylhydrazone, which dissipate the transmembrane proton gradient. Treatment with either compound significantly suppressed ENT3-AA-mediated [^14^C]urate uptake at pH 5.0 (Figure 2B), indicating that ENT3-AA-mediated transport depends on an inwardly directed proton gradient. These findings support a proton-coupled mechanism for urate export by ENT3 in the lysosome, where it can use an outwardly directed proton gradient from the acidic environment in the lysosomal lumen to the near neutral cytosol. Thus, the pH dependence is not likely a reflection of the equilibrative transport of the increasing fraction of the unionized form of urate with decreasing pH.

Next, we tested whether ENT3-AA-mediated urate transport is influenced by other ions by replacing NaCl in the uptake buffer with equimolar concentrations of KCl, Na-gluconate, K-gluconate, or mannitol. As shown in Figure 2C, substitution with K^+^ or mannitol had no effect on ENT3-AA-mediated [^14^C]urate uptake, suggesting that ENT3 function is independent of Na^+^, K^+^, or Cl^−^. A modest reduction in uptake was observed when Cl^−^ was replaced with gluconate, but not when NaCl was replaced with mannitol, implying that the effect likely reflects weak inhibition by gluconate rather than a requirement for Cl^−^.

Time-course analysis revealed that [^14^C]urate uptake in ENT3-AA-transfected cells increased proportionally to time over the initial 2 min of incubation period (Figure 2D). [^14^C]Urate uptake in mock-transfected cells was significantly lower, confirming the activity of ENT3-AA in its transfected cells, and was also increased proportionally to time over the same initial period. Based on these results, the 2-min incubation period used in regular uptake assays was confirmed to be suitable for evaluation of transport in the initial phase.

Kinetic analysis demonstrated that ENT3-AA-mediated [^14^C]urate uptake followed Michaelis-Menten kinetics, displaying a saturable profile with the maximum transport rate (*V*_max_) of 2.65 nmol/min/mg protein and the Michaelis constant (*K*_m_) of 1.15 mM (Figure 2E). Notably, this *K*_m_ value exceeds the saturated level of urate concentration (∼0.4 mM) that may occur in the presence of phagocytosed MSU crystals in lysosomes, suggesting that ENT3 is capable of mediating lysosomal urate efflux efficiently even under such conditions. These data support the characterization of ENT3 as a low-affinity, high-capacity urate transporter. The [^14^C]urate concentrations at high levels, which were achieved by dissolving urate in alkaline buffer first and subsequently adjusting pH to the acidic condition, were used in this analysis for the evaluation of transport under the conditions relevant to lysosomal urate clearance and inflammatory regulation within macrophages.

We then attempted to further explore the characteristics of ENT3 for the recognition of substrates and/or inhibitors. In the attempt, we first assessed the effects of specific ENT inhibitors, dipyridamole and nitrobenzylthioinosine (NBMPR),^14^^,^^17^^,^^18^ on the ENT3-AA-mediated uptake of [^14^C]urate (4 μM). The ENT3-AA-mediated uptake was inhibited by both compounds in a concentration-dependent manner, with the half-maximal inhibitory concentration (*IC*_50_) values of 7.71 μM and 21.7 μM, respectively (Figure 2F). These were comparable to those of 2.8 μM and 14.7 μM, respectively, for ENT2-mediated urate transport determined in our previous study.^13^ We then tested a panel of nucleosides, nucleobases, and small molecules for their inhibitory action. As shown in Figure 2G, no significant inhibition was observed for any nucleoside or nucleobase at the concentration (0.1 mM for guanine and 1 mM for the others) comparable to their physiological concentrations in lysosomes. Notably, adenosine inhibited the ENT3-AA-mediated uptake moderately only at a high concentration (10 mM), consistent with the reported low affinity of ENT3 for this substrate (*K*_m_ = 1.86 mM).^14^ Given that lysosomal adenosine concentrations could be not higher than ∼3 mM,^19^ the impact of its inhibitory action is likely to be minimal physiologically. Among the anionic compounds tested (0.1 mM), urate-lowering agents, such as febuxostat, benzbromarone, and probenecid, and anti-inflammatory drugs commonly used for gout management, such as diclofenac, indomethacin, and phenylbutazone, inhibited the ENT3-AA-mediated uptake moderately by 20%–60%. However, given that the therapeutic plasma concentrations of these drugs typically remain below 0.1 mM,^20^^,^^21^^,^^22^ their inhibitory effects on ENT3 are likely to be minimal in clinical situations. In addition, estrone-3-sulfate, bumetanide, empagliflozin, furosemide, and losartan also exhibited moderate inhibitory effects.

Collectively, these findings establish ENT3 as a proton-coupled urate transporter that operates optimally under acidic conditions and exhibits kinetic characteristics consistent with its proposed role in lysosomal urate efflux. The high *K*_m_ value (1.15 mM) relative to intralysosomal urate concentrations supports its classification as a low-affinity, high-capacity transporter. Furthermore, the relative insensitivity of ENT3 to endogenous nucleosides, nucleobases, and clinically relevant urate-lowering agents underscores its distinct substrate and inhibitor selectivity. These mechanistic insights strengthen the understanding of the role of ENT3 in regulating lysosomal urate homeostasis.

### Impact of ENT3 genetic variants on urate transport

Multiple pathogenic variants in the *SLC29A3* gene, which encodes ENT3, have been linked to several rare inherited disorders, including H syndrome,^23^^,^^24^^,^^25^^,^^26^^,^^27^^,^^28^ Rosai-Dorfman disease,^23^^,^^24^^,^^28^ pigmentary hypertrichosis with non-autoimmune insulin-dependent diabetes mellitus (PHID),^23^^,^^24^^,^^25^^,^^28^ sinus histiocytosis with massive lymphadenopathy,^23^ and dysosteosclerosis.^23^ These disorders are characterized by immune dysregulation, and loss-of-function mutations in ENT3 are thought to play a central role in their pathogenesis, although detailed mechanisms, including specific ENT3 substrates involved, have not been fully clarified yet.

**Figure 3:**
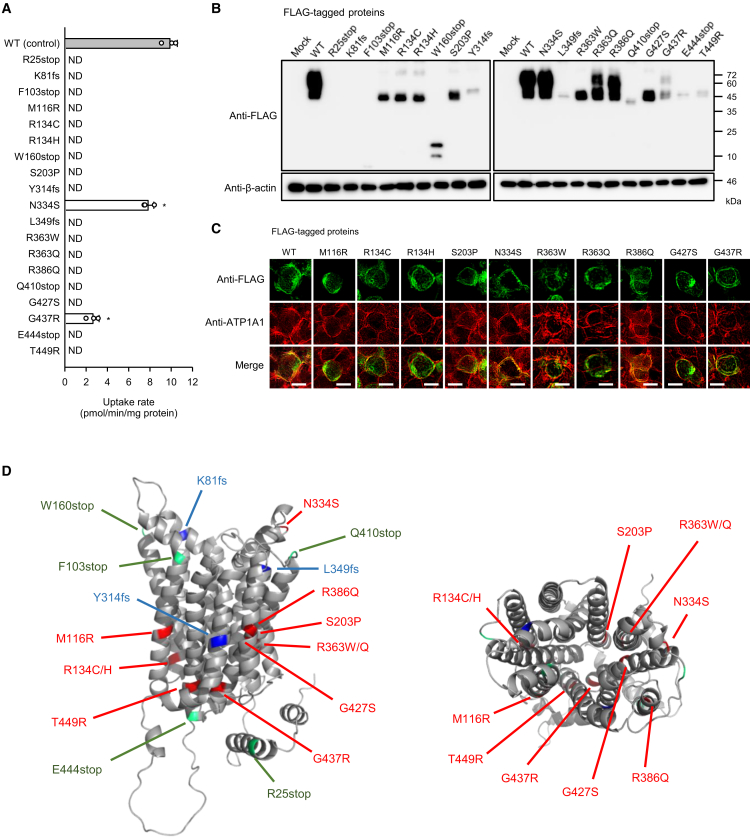
Effects of genetic mutations on urate transport by ENT3-AA (A) Uptake of [^14^C]urate (4 μM) by FLAG-tagged ENT3-AA (WT) and its mutants was evaluated over 2 min at pH 5.0 and 37°C in transiently transfected HEK293 cells. ND, not detected. (B) Western blotting was performed using anti-FLAG antibody on whole-cell lysates (10 μg protein per lane) prepared from transiently transfected HEK293 cells. β-actin blots are shown as loading controls. (C) Immunofluorescent imaging revealed the co-localization of FLAG-ENT3-AA (WT) and its mutants (green) with ATP1A1 (red), a plasma membrane marker, in transiently transfected HEK293 cells. Scale bars: 10 μm (D) Structural model of ENT3 was visualized using PyMOL based on AlphaFold predictions.^29^ The left and right panels show horizontal and extracellular side views, respectively. Amino acid residues altered by missense mutations, stop codon insertions, and frameshift mutations are shown in red, green, and blue, respectively. Data are presented as the mean ± SD from three biological replicates using separate cell preparations. (A) Statistical analysis was performed using one-way ANOVA followed by Dunnett’s test. ^∗^*p* < 0.05 versus control.

To assess the impact of disease-associated ENT3 mutations on the newly identified urate transport function, we generated a panel of FLAG-tagged ENT3-AA mutants harboring previously reported *SLC29A3* variants (Table S1) and evaluated their capability to transport urate in transiently transfected HEK293 cells. As shown in Figure 3A, urate transport activity represented by the specific uptake of [^14^C]urate (4 μM) by each mutant was completely abolished in all mutants except for N334S and G437R, which exhibited significantly reduced—but still detectable—transport activity relative to the wild type (WT) of ENT3-AA. Western blot analysis using the anti-FLAG antibody demonstrated that the expression levels of N334S, R363Q, and R386Q were comparable to that of ENT3-AA (Figure 3B), whereas the other mutants—particularly those bearing stop codon insertion or frameshift mutations (R25stop, K81fs, F103stop, W160stop, Y314fs, L349fs, Q410stop, and E444stop)—exhibited markedly reduced or undetectable expression. To validate these observations, we introduced the same mutations into FLAG-tagged WT ENT3 and examined their expression profiles. As shown in Figure S1, they were similar to those of ENT3-AA mutants, suggesting that certain mutations impair protein stability or translational efficiency. Immunofluorescence analysis further revealed that a subset of ENT3-AA mutants with detectable expression (M116R, R134C, R134H, S203P, N334S, R363W, R363Q, R386Q, G427S, and G437R) localized to the plasma membrane in a manner similar to WT ENT3-AA (Figure 3C), indicating that their reduced or lost transport activity is not attributable to mislocalization. Among these, the N334S mutant showed reduced urate transport despite normal expression and localization, implying a direct effect on the transport function (substrate recognition and/or translocation). Likewise, mutants such as M116R, R134C, R134H, S203P, R363W, R363Q, R386Q, and Q427S maintained membrane localization but failed to transport urate, indicating a complete loss of transport activity. In contrast, the G437R mutant displayed partial activity that appeared to correlate with reduced protein expression, suggesting that transport function might not be impaired.

Structural analysis based on the AlphaFold2-predicted ENT3 model (Figure 3D) provides further mechanistic insight into the impact of disease-associated variants. The amino acid residues with stop codon insertion or frameshift mutations, which caused markedly reduced or undetectable expression presumably due to global disruption of protein translation or stability, are distributed throughout the protein in predicted transmembrane helices and other regions. In contrast, the residues with missense mutations, which were suggested to lead to impaired transport function, cluster within predicted transmembrane helices, which generally play major roles in transport function, in accordance with the suggested functional impairment.

These results underscore the functional repertoire of ENT3 extended beyond nucleoside transport, highlighting its role in urate handling, and the need to further investigate ENT3 variants in the context of urate-associated inflammatory and metabolic disorders.

### Role of ENT3 in modulating urate accumulation and the inflammatory response in differentiated THP-1 cells

**Figure 4:**
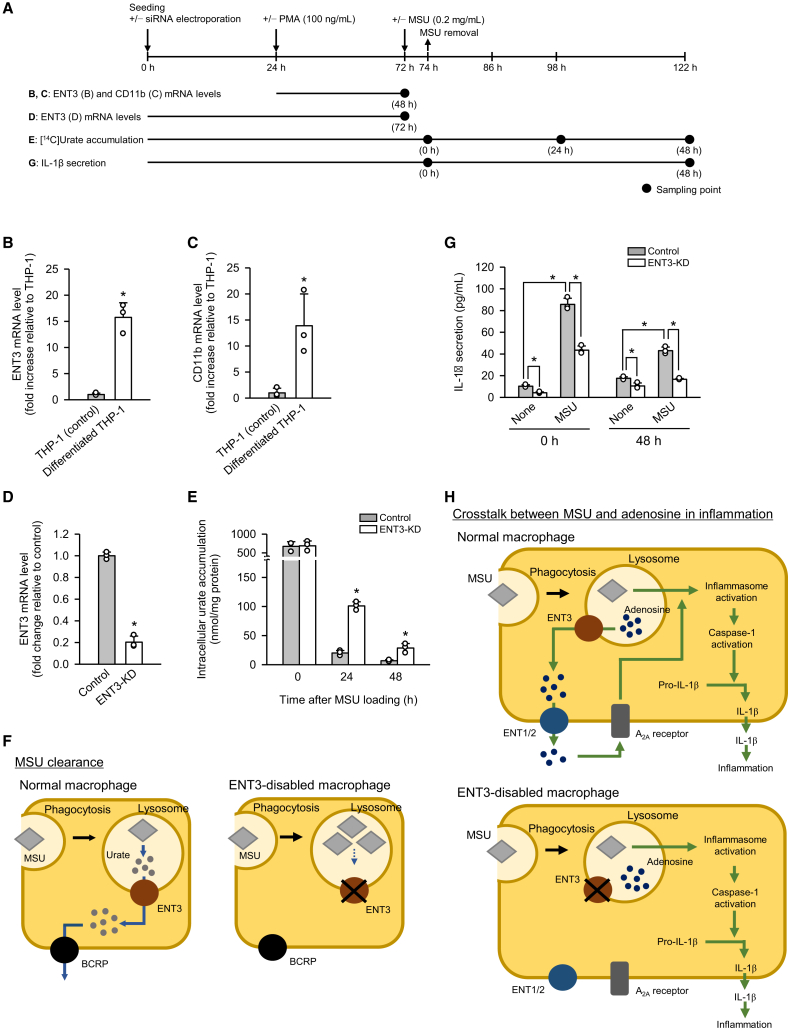
Effect of knockdown of endogenous ENT3 on accumulation of MSU-derived urate and inflammation in differentiated THP-1 cells (A) Schematic overview of the experimental timelines. (B and C) mRNA levels of ENT3 and CD11b were quantified by real-time PCR in undifferentiated and differentiated THP-1 cells. (D) Real-time PCR analysis of ENT3 mRNA levels in differentiated control and ENT3-knocked down (ENT3-KD) THP-1 cells. (E) Accumulation of [^14^C]urate derived from [^14^C]MSU was assessed in differentiated control and ENT3-KD THP-1 cells at 0, 24, and 48 h following 2-h loading of [^14^C]MSU (0.2 mg/mL). (F) Proposed model of ENT3 function for lysosomal urate elimination. (G) IL-1β concentrations in culture supernatants were quantified by ELISA at 0 and 48 h following 2-h MSU loading (0.2 mg/mL). (H) Proposed model of ENT3 function for exporting adenosine across the lysosomal membrane to regulate inflammatory responses. ENT3 dysfunction leads to suppression of adenosine-dependent inflammasome activation. Data are presented as the mean ± SD from three biological replicates using separate cell preparations. Statistical significance was evaluated using Student’s *t* test (B–E) or two-way ANOVA followed by Bonferroni post-hoc test (G). ^∗^*p* < 0.05 versus control (B–E) or as indicated (G).

We examined whether ENT3 knockdown modulates urate accumulation and the inflammatory response to MSU crystals, a principal driver of gout-related inflammation, in differentiated THP-1 cells as a macrophage-like model, following the experimental procedures outlined in Figure 4A. Although LPS is generally used as a priming signal for IL-1β induction, we did not use it as we intended to examine the role of ENT3 more directly. We first assessed ENT3 expression during the differentiation of THP-1 cells into the macrophage-like status from the monocyte status by the treatment with phorbol 12-myristate 13-acetate (PMA, 100 ng/mL) for 48 h. Notably, ENT3 mRNA expression was significantly upregulated following differentiation (Figure 4B), suggesting its preferential expression in macrophages. In parallel, expression of CD11b mRNA—a well-established marker of macrophage differentiation^30^—was also elevated (Figure 4C), confirming the phenotypic transition. These findings implicate the role of ENT3 in macrophage-specific physiological processes.

We next examined the effects of ENT3 knockdown, which was confirmed to reduce ENT3 mRNA expression effectively (Figure 4D), in differentiated THP-1 cells. To evaluate the role of ENT3 in lysosomal urate handling, we measured intracellular [^14^C]urate levels after exposing differentiated THP-1 cells to [^14^C]MSU (0.2 mg/mL) for 2 h for its phagocytosis, and then cultured for an additional 24 or 48 h in its absence. As shown in Figure 4E, while the levels of [^14^C]urate accumulation were comparable between control and ENT3-knocked down (ENT3-KD) cells initially at 0 h after the removal of [^14^C]MSU, they were higher in ENT3-KD cells at 24 and 48 h, indicating the impairment of urate clearance by ENT3 knockdown. These findings indicate that ENT3 facilitates the efflux of urate derived from phagocytosed MSU across the lysosomal membrane (Figure 4F). By maintaining low intralysosomal urate concentrations, ENT3 may promote MSU solubilization and prevent sustained lysosomal stress, which leads to inflammatory response. Based on this, ENT3 knockdown could enhance inflammatory response by delaying lysosomal urate elimination. The cellular uptake and handling of [^14^C]MSU was not possible to assess in the model system using transiently transfected HEK293 cells because of their only limited capability for phagocytosis.

The inflammatory response represented by IL-1β secretion into the culture supernatant was, however, suppressed by ENT3 knockdown. As shown in Figure 4G, when assessed at the initial time point (0 h) after 2-h exposure of the cells to MSU (0.2 mg/mL), IL-1β secretion was increased in both control and ENT3-KD cells by the exposure to MSU, but to a lesser extent in the latter. IL-1β secretion decreased with time in cells exposed to MSU, but it was still greater in control cells than in ENT3-KD cells at 48 h. The knockdown of ENT3 likely reduced cytosolic adenosine levels and thereby limited adenosine-induced inflammatory signaling (Figure 4H), as adenosine has been shown to potentiate NLRP3 inflammasome activation via A_2A_ receptor and thereby promote a stronger and more sustained IL-1β secretion, although it does not activate the inflammasome by itself.^15^ Thus, the hypothesized enhancement of inflammatory response by ENT3 knockdown was not observed presumably due to counteracting suppression of adenosine-induced inflammatory signaling. It could also be possible alternatively that delayed MSU clearance itself may trigger compensatory mechanisms in inflammasome signaling, which could contribute to the observed reduction of IL-1β secretion. This issue remains to be investigated in more detail in the future. Particularly, the analysis of IL-1β section in a time-dependent manner could be of help for more detailed and comprehensive understanding.

Taken together, our findings position ENT3 as a critical regulator of lysosomal MSU-induced and adenosine-dependent inflammation in macrophages. Dysfunction of ENT3 may contribute to the pathogenesis of gout and related disorders not only by impairing lysosomal urate clearance, leading to sustained MSU crystal retention and lysosomal stress for inflammasome activation, but also by disrupting lysosomal adenosine efflux, leading to the suppression of inflammasome activation. This highlights a dual contribution of ENT3 to inflammation: it mitigates MSU-induced lysosomal damage and simultaneously facilitates adenosine-dependent inflammasome activation. These findings suggest that ENT3 acts as a physiological regulator that limits excessive MSU-induced inflammation while allowing appropriate immune activation via the adenosine pathway.

A recent study by Cobo et al. demonstrated that lysosomal damage and inflammatory signaling are, while interconnected, governed by distinct transcriptional programs in macrophages during particle-induced inflammation.^31^ This report could broaden the pathological framework involving ENT3 function and further underscore the relevance of our findings. While excessive inflammation may cause tissue damage, complete immune suppression can impair host defense. Thus, ENT3 may help balance inflammatory responses to ensure proper resolution and maintain tissue homeostasis.

Notably, it is conceivable that, as shown in Figure 4F, urate exported from lysosomes via ENT3 is subsequently extruded from macrophages by the breast cancer resistance protein (BCRP/ABCG2), which has been implicated in urate efflux in a previous study.^32^ Given this sequential pathway, impaired BCRP function may lead to cytosolic urate accumulation and thereby delay lysosomal urate elimination. BCRP plays the major role in systemic urate elimination, and loss-of-function mutations in *ABCG2* have been linked to hyperuricemia and gout.^33^^,^^34^ In macrophages, coordinated operation of ENT3 and BCRP may be needed for urate elimination and its impairment may lead to local inflammation and disease progression.

### Conclusion

This study has identified a novel role for ENT3 as a proton-coupled urate transporter localized to lysosomes. Our data have demonstrated that ENT3 mediates urate efflux and thereby reduce lysosomal urate accumulation. This could potentially limit MSU crystal persistence and attenuate inflammasome activation in macrophages. In addition, concurrent ENT3-mediated adenosine efflux may oppositely play a role in the enhancement of inflammasome activation, underscoring its broader role in immune regulation. These findings extend the functional repertoire of ENT3 beyond nucleoside transport and highlight its potential as a therapeutic target for gout and other urate-associated inflammatory conditions.

### Limitations of the study

These conclusions are, however, primarily based on experiments conducted in transient overexpression systems using HEK293 cells, which may not fully recapitulate the physiological behavior of ENT3 in primary macrophages or other pathophysiologically relevant cell types under endogenous expression levels. Moreover, the *in vivo* relevance of ENT3-mediated lysosomal urate transport remains to be established, as global Ent3 knockout mice exhibit severe postnatal abnormalities and early mortality,^35^ limiting their utility for studying urate disposition under physiological or disease-relevant conditions, and suitable conditional knockout models that allow tissue-specific or inducible deletion of Ent3 in the context of hyperuricemia have yet to be developed. In addition, our kinetic analyses were performed using a plasma membrane-relocated ENT3-AA construct and, hence, may not precisely reflect the native properties of WT ENT3 within the acidic lysosomal environment. Future directions should include validation in macrophage-specific Ent3 knockout models and/or MSU-induced *in vivo* inflammatory models, and the identification of specific ENT3 inhibitors that could allow functional interrogation. Particularly, studies employing macrophage-specific Ent3 knockout models, along with direct measurements of urate transport in lysosomes using organelle-targeted urate sensors—tools that are not yet available—would be important for validating and further elucidating the physiological and pathological relevance of the findings in this study.

## Resource availability

### Lead contact

Further information and requests for resources should be directed to and will be fulfilled by the lead contact, Tomoya Yasujima (yasujima@phar.nagoya-cu.ac.jp).

### Materials availability

This study did not generate new unique reagents.

### Data and code availability

• All data supporting the findings of this study are included within the article and its supplemental information, or are available from the corresponding author upon reasonable request.
• This study did not generate any code.
• Any additional information required to reanalyze the data reported in this paper is available from the corresponding author upon request.

## Acknowledgments

This study was supported in part by JSPS 10.13039/501100001691KAKENHI (grant number JP20K07135), a 2019 research grant from the 10.13039/100008732Uehara Memorial Foundation, and a 2023 research grant from the 10.13039/501100005865Mochida Memorial Foundation for Medical and Pharmaceutical Research.

## Author contributions

Conceptualization, T. Yasujima and I.M.; formal analysis, T. Yasujima, I.M., H.M., A.N., Y.T., T.T., K.I., and T. Yamashiro; investigation, I.M.; visualization, T. Yasujima and I.M.; writing – original draft preparation, T. Yasujima and I.M.; writing – review and editing, H.M., A.N., Y.T., T.T., K.I., and H.Y.; supervision, H.Y.; project administration, T. Yasujima; funding acquisition, T. Yasujima; all authors read and approved the final manuscript.

## Declaration of interests

The authors declare no competing interests.

## STAR★Methods

### Key resources table

**Table:** 

REAGENT or RESOURCE	SOURCE	IDENTIFIER
**Antibodies**		

Anti-FLAG (1:1000)	FUJIFILM Wako	Cat#014–22383; RRID: AB_10659717
Anti-β-actin (1:1000)	Proteintech	Cat#60008-1-Ig; RRID: AB_2289225
Anti-HA (1:1000)	Medical & Biological Laboratories	Cat#M180-3; RRID: AB_10951811
Goat anti-mouse IgG secondary antibody, HRP (1:10000)	Jackson ImmunoResearch	Cat#115-035-003; RRID: AB_10015289
Rabbit polyclonal anti-ATP1A1 (1:500)	Proteintech	Cat#55187-1-AP; RRID: AB_10859261
Goat anti-mouse IgG secondary antibody, DyLight 488 (1:500)	SeraCare Life Sciences	Cat#042-03-18-06; RRID: not provided
Goat anti-rabbit IgG secondary antibody, Alexa Fluor 594 (1:500)	Jackson ImmunoResearch	Cat#111-585-003; RRID: AB_2338059

**Bacterial and virus strains**		

OmniMax 2 T1R	Thermo	Cat#C854003

**Chemicals, peptides, and recombinant proteins**		

[^14^C]Urate	American Radiolabeled Chemicals	Cat#0513A
[^3^H]Adenosine	American Radiolabeled Chemicals	Cat#0287A
DMEM	FUJIFILM Wako	Cat#043-30085
RPMI 1640	FUJIFILM Wako	Cat#189-02025
Opti-MEM	Thermo	Cat#31985070
FBS	Sigma-Aldrich	Cat#173012
Penicillin/streptomycin	FUJIFILM Wako	Cat#168-23191
Polyethylenimine	Polysciences	Cat#24765
ReverTra Ace	Toyobo	Cat#TRT-101
KOD ONE	Toyobo	Cat#KMM-101
QuantumDye v3.1	Digital Biology	Cat#QSD3
Hi-Di Formamide	Thermo	Cat#4401457
MES	Dojindo	Cat#345-01625
HEPES	Dojindo	Cat#H0396
BSA	FUJIFILM Wako	Cat#015-27053
Taq Pro Universal SYBR qPCR Master Mix	Vazyme	Cat#Q712-02
Tris	FUJIFILM Wako	Cat#207-06275
SDS	FUJIFILM Wako	Cat#194-13985
urea	FUJIFILM Wako	Cat#217-00615
EDTA	DOJINDO	Cat#345-01865
NaCl	FUJIFILM Wako	Cat#191-01665
KCl	FUJIFILM Wako	Cat#163-03545
Na-gluconate	FUJIFILM Wako	Cat#193-13195
K-gluconate	FUJIFILM Wako	Cat#169-11835
Mannitol	FUJIFILM Wako	Cat#133-00845
Luminate Forte Western HRP Substrate	Merck Millipore	Cat#WBLUF0100
Triton X-	Nakarai Tesque	Cat#28229-25
PMA	Sigma-Aldrich	Cat#P1585
PVDF membrane	Merck Millipore	Cat#IPVH00010
CCCP	FUJIFILM Wako	Cat#038-16991
FCCP	TOKYO CHEMICAL	Cat#C3463
Urate	Sigma-Aldrich	Cat#U2625
Adenosine	FUJIFILM Wako	Cat#010-10491
Guanosine	FUJIFILM Wako	Cat#079-0111
Thymidine	FUJIFILM Wako	Cat#205-08091
Cytidine	FUJIFILM Wako	Cat#039-05333
Uridine	FUJIFILM Wako	Cat#213-00771
Inosine	FUJIFILM Wako	Cat#099-00231
Adenine	Sigma-Aldrich	Cat#A8626
Guanine	FUJIFILM Wako	Cat#079-01091
Thymine	FUJIFILM Wako	Cat#205-01391
Cytosine	Sigma-Aldrich	Cat#C3506
Uracil	FUJIFILM Wako	Cat#210-0063
Hypoxanthine	FUJIFILM Wako	Cat#088-03402
Xanthine	Sigma-Aldrich	Cat#X0626
Dipyridamole	Sigma-Aldrich	Cat#D9766
NBMPR	FUJIFILM Wako	Cat#323-34731
DIDS	Cayman	Cat#16125
Estrone-3-sulfate	Sigma-Aldrich	Cat#E9145
Methotrexate	FUJIFILM Wako	Cat#139-13571
Nicotinic acid	FUJIFILM Wako	Cat#142-01232
PAH	Cayman	Cat#23726
PZA	FUJIFILM Wako	Cat#168-13642
Allopurinol	Sigma-Aldrich	Cat#A8003
Febuxostat	TOKYO CHEMICAL	Cat#F0847
Benzbromarone	Sigma-Aldrich	Cat#B5774
Probenecid	Sigma-Aldrich	Cat#P8761
Sulfinpyrazone	Sigma-Aldrich	Cat#S9509
Colhicine	FUJIFILM Wako	Cat#039-03851
Diclofenac	Sigma-Aldrich	Cat#043-22851
Indomethacin	Nakarai Tesque	Cat#192-33
Phenylbutazone	FUJIFILM Wako	Cat#P319570
Salicylic acid	FUJIFILM Wako	Cat#199-00142
Bumetanide	Sigma-Aldrich	Cat#B-3023
Empagliflozin	Cayman	Cat#17375
Furosemide	Sigma-Aldrich	Cat#F-4381
Losartan	Cayman	Cat#10006594

**Critical commercial assays**		

Protein Assay BCA Kit	FUJIFILM Wako	Cat#297-73101
Human IL-1 beta ELISA kit	Proteintech	Cat#KE00021

**Experimental models: Cell lines**		

THP-1	JCRB	Cat#JCRB112.1
HEK293	RIKEN BRC	Cat#RCB1637

**Oligonucleotides**		

Oligo(dT)20	Sigma-Aldrich	N/A
Primers for amplification of the cDNA for ENT3 in Table EV2	Sigma-Aldrich	N/A
Negative control Silencer-Selected siRNA	ThermoFisher	Cat#4390844
Primers for the generation of the cDNAs for ENT3-AA in Table EV3	Sigma-Aldrich	N/A
Primers for the generation of the cDNAs for ENT3 variants in Table EV4	Sigma-Aldrich	N/A
Primers for the real-time PCR analyses of the expression in Table EV5	Sigma-Aldrich	N/A
siRNAs for human ENT3 in Table EV6	ThermoFisher	N/A
siRNA sequences are listed in Table S6	This paper	N/A

**Recombinant DNA**		

pCI-neo	Promega	Cat#E1841

**Software and algorithms**		

WinNonlin	Certara	https://www.certara.com/software/phoenix-winnonlin/
SigmaStat	Grafiti	https://grafiti.com/sigmastat/

### Experimental model and study participant details

#### Cell lines

##### HEK293 cells

HEK293 cells were obtained from the Cell Resource Center for Biomedical Research, Tohoku University (Sendai, Japan), and cultured in Dulbecco’s Modified Eagle Medium (Thermo Fisher Scientific, Waltham, MA, USA) supplemented with 10% fetal bovine serum (FBS; Sigma-Aldrich, St. Louis, MO, USA), 100 U/mL penicillin, and 100 μg/mL streptomycin. Cells were maintained at 37 °C in a humidified atmosphere containing 5% CO_2_. Cells were authenticated by the provider using short tandem repeat profiling. Cells were routinely tested and confirmed to be free of mycoplasma contamination.

##### THP-1 cells

THP-1 cells were obtained from the Japanese Collection of Research Bioresources Cell Bank (Osaka, Japan) and cultured in RPMI 1640 medium (Thermo Fisher Scientific) supplemented with 10% heat-inactivated FBS, 100 U/mL penicillin, and 100 μg/mL streptomycin. THP-1 cells were authenticated by JCRB using STR analysis and were confirmed to be mycoplasma-negative. For differentiation into macrophage-like cells, THP-1 cells were treated with 100 ng/mL PMA (Sigma-Aldrich) for 48 h.

### Method details

#### Preparation of plasmids

Human ENT3 cDNA (GenBank accession number: NM_018344.6) was cloned via reverse transcription-PCR (RT-PCR), following the method previously reported,^36^ with minor modifications. Total RNA extracted from human macrophages (BioChain Institute, Newark, CA, USA) was used as the template. Reverse transcription was carried out, using 1 μg of total RNA, an oligo(dT) primer, and ReverTra Ace reverse transcriptase (Toyobo, Osaka, Japan), to generate a cDNA mixture. The ENT3 coding region was then amplified using KOD One DNA Polymerase (Toyobo). A second PCR was performed using the initial PCR product as a template to introduce restriction enzyme sites for subcloning. Because the Xba I site is located near the stop codon of ENT3, the reverse primer used in the first PCR was reused in the second amplification step. Primer sequences used for these procedures are provided in Table S2. The ENT3-AA cDNA was created through site-directed mutagenesis using KOD One DNA Polymerase and specific primers listed in Table S3. Produced cDNAs for ENT3 and ENT3-AA were inserted into the pCI-neo expression vector (Promega, Madison, WI, USA), and sequence verification was conducted using an ABI PRISM 3130 DNA sequencer (Applied Biosystems, Foster City, CA, USA), in accordance with standard procedures.

To construct N-terminal FLAG-tagged versions of ENT3 and ENT3-AA, the corresponding cDNA fragments were inserted into a modified pCI-neo vector that contains an N-terminal FLAG epitope sequence. Site-directed mutagenesis was also employed to generate cDNAs for various ENT3-AA mutants. These constructs were prepared using the PrimeSTAR Mutagenesis Basal Kit (Takara Bio, Kusatsu, Japan) along with KOD One DNA Polymerase and inserted into the modified pCI-neo vector for FLAG attachment. The primers used for mutagenesis are listed in Table S4.

Additionally, the plasmid for HA-tagged SNBT1 (GenBank accession number: NM_001270038.1) was prepared using the pCI-neo vector, according to the method previously reported.^16^

#### Transient transporter expression in HEK293 cells

HEK293 cells were seeded at a density of 2.0 × 10^5^ cells per well in 24-well culture plates pre-coated with poly-L-lysine and allowed to adhere for 12 h. Transient transfection was performed by introducing 1 μg of plasmid DNA encoding the target transporter into each well, using 2 μg of polyethylenimine “MAX” (Polysciences, Warrington, PA, USA) as the transfection reagent. After transfection, the cells were maintained under standard culture conditions for an additional 48 h to allow for protein expression.

In experiments to examine lysosomal urate accumulation using cells transfected with HA-SNBT1, with or without FLAG-ENT3, the cells were cultured in 6-well plates. The amount of plasmid DNA encoding the transporter was, when required, partially substituted with the empty pCI-neo vector to ensure that the total amount remained constant across experimental groups.

#### Transport study

Uptake experiments were performed using transiently transfected HEK293 cells seeded into poly-L-lysine-coated 24-well plates, following a previously established protocol with minor modifications (Mimura et al., 2017). The uptake buffer was based on Hanks’ balanced salt solution, supplemented with 10 mM MES for acidic conditions (pH ≤ 6.5) or 10 mM HEPES for neutral or alkaline conditions (pH ≥ 7.0). Radiolabeled [^14^C]urate or [^3^H]adenosine was included as the test substrate.

Prior to initiating the uptake, cells were preincubated at 37 °C for 5 min in substrate-free buffer. Uptake was initiated by replacing the buffer with substrate-containing solution and allowed to proceed for a defined time at 37 °C. To assess the impact of ionic composition, NaCl in the uptake buffer was selectively replaced with alternative components as specified in each experimental condition. For inhibition assays, test compounds were included only during the uptake phase. To terminate uptake at the end of the specified period, the solution was removed, and cells were washed and lysed. Radioactivity associated with the cell lysate was measured using liquid scintillation counting. Protein content in each well was quantified using the bicinchoninic acid (BCA) method (BCA Protein Assay Reagent Kit; FUJIFILM Wako Pure Chemical, Osaka, Japan), with bovine serum albumin (BSA) used as the standard. Specific uptake was calculated by subtracting the uptake measured in mock cells from that in transporter-expressing cells.

#### Isolation of lysosomal fractions

Lysosomal fractions were prepared from transiently transfected HEK293 cells after [^14^C]urate uptake, according to a modified differential centrifugation method based on a previously published protocol.^37^ Cells cultured in 100 mm dishes were harvested and suspended in an ice-cold homogenization buffer composed of 50 mM mannitol and 20 mM HEPES (pH 7.4). The suspension was homogenized using a glass-Teflon homogenizer under chilled conditions. The homogenate was subjected to centrifugation at 1,500 × g for 10 min at 4 °C to remove nuclei, cytoskeletal elements, and mitochondria. The resulting supernatant was further centrifuged at 24,000 × g for 30 min at 4 °C, and the pellet obtained was collected as the lysosome-enriched fraction. All procedures were carried out at 4 °C to preserve organelle integrity.

#### Western blot analysis

HEK293 cells were transiently transfected with expression plasmids encoding FLAG-ENT3, FLAG-ENT3-AA, various FLAG-ENT3-AA mutants, or HA-SNBT1, or co-transfected with HA-SNBT1 and FLAG-ENT3. Following a 48-h culture, cells were washed three times with ice-cold phosphate-buffered saline (PBS, pH 7.4) and lysed in ice-cold lysis buffer (pH 7.4) containing 4 M urea, 150 mM NaCl, 50 mM Tris-HCl, 1 mM EDTA, and 1% SDS. Lysates were centrifuged at 24,000 × g for 10 min at 4 °C, and the supernatant was mixed with 4 × SDS sample buffer (pH 6.8) composed of 40% glycerol, 240 mM Tris-HCl, 8% SDS, 5% 2-mercaptoethanol, and 0.04% bromophenol blue.

Protein samples were separated on 15% SDS-polyacrylamide gels and transferred to PVDF membranes (Immobilon-P; Merck Millipore, Burlington, MA). Membranes were blocked with 5% skim milk in Tris-buffered saline (TBS) containing 0.1% Tween 20 (TBS-T) and then incubated overnight at 4 °C with either mouse anti-FLAG monoclonal antibody (FUJIFILM Wako Pure Chemical) or mouse anti-HA monoclonal antibody (Medical & Biological Laboratories, Nagoya, Japan), both diluted 1:1,000 in TBS-T. After three washes with TBS-T, membranes were incubated for 1 h at room temperature with horseradish peroxidase-conjugated goat anti-mouse IgG (Jackson ImmunoResearch, West Grove, PA, USA) diluted 1:20,000. To detect β-actin, mouse anti-β-actin monoclonal antibody (Proteintech, Rosemont, IL, USA) was used at 1:1,000 dilution under the same conditions as described. Signal detection was performed using enhanced chemiluminescence and visualized with the ChemiDoc Touch Imaging System (Bio-Rad Laboratories, Hercules, CA).

#### Immunofluorescence analysis

Immunofluorescence staining was performed based on a previously described protocol,^36^ with minor modifications. HEK293 cells transiently expressing target proteins were washed three times with PBS (pH 7.4), fixed, and permeabilized in 100% methanol for 20 min at −20°C. After additional PBS washes, the cells were incubated with PBS containing 5% BSA for 1 h at room temperature to block non-specific binding.

Primary antibodies—mouse anti-FLAG monoclonal antibody, mouse anti-HA monoclonal antibody, and rabbit anti-ATP1A1 polyclonal antibody (Proteintech)—were applied at a dilution of 1:500 in PBS with 1% BSA and incubated for 1 h at room temperature. Following washing, cells were incubated for 1 h with DyLight 488-conjugated goat anti-mouse IgG (Kirkegaard & Perry Laboratories, Gaithersburg, MD, USA) and Alexa Fluor 594-conjugated goat anti-rabbit IgG (Jackson ImmunoResearch) as secondary antibodies, each diluted in PBS. Fluorescent images were captured using an all-in-one fluorescence microscope (BZ-X810; KEYENCE, Osaka, Japan).

#### Quantification of ENT3 mRNA by real-time PCR

Total RNA was extracted from THP-1 cells using RNAiso Plus reagent (FUJIFILM Wako Pure Chemical) according to the guanidine isothiocyanate-phenol-chloroform method.^38^ cDNA was obtained from 1 μg of total RNA using ReverTra Ace reverse transcriptase with oligo(dT) primers. Quantitative real-time PCR was performed using a CFX Connect Real-Time PCR Detection System (Bio-Rad Laboratories) and Taq Pro Universal SYBR qPCR Master Mix (Nanjing Vazyme Biotech, Nanjing, China). Primer sequences specific for ENT3 and the internal control gene GAPDH are listed in Table SV5. The relative level of ENT3 mRNA expression was determined by normalizing to GAPDH mRNA expression using the ΔΔCt method.

#### Knockdown of ENT3 in THP-1 cells

For knockdown of ENT3, THP-1 cells were suspended in Opti-MEM (Thermo Fisher Scientific) at a density of 5.0 × 10^6^ cells per cuvette, and 200 pmol of siRNA was added to each cell suspension for electroporation using the NEPA21 Type II system (Nepa Gene, Chiba, Japan). Following electroporation, cells were immediately transferred to RPMI 1640 medium, and non-viable cells were removed. The viable cells were then seeded into 24-well plates for experiments to assess intracellular urate accumulation and 6-well plates for the other experiments. On the following day, PMA (100 ng/mL) was added, and the cells were cultured for 48 h to induce differentiation into macrophage-like cells. Sequences of siRNAs used in this study are listed in Table S6. A non-targeting negative control siRNA was used as a control.

#### Preparation of MSU

MSU crystals were generated by a modified recrystallization method based on a previous report.^39^ Briefly, 336 mg of urate was dissolved in 80 mL of 25 mM NaOH and heated to 80 °C. After cooling to room temperature, the solution was filtered through a 0.2 μm membrane and stored at 4 °C for 4 days, allowing crystal formation. The resulting MSU crystals were collected by centrifugation at 24,000 × g for 1 min and resuspended in PBS. To prepare [^14^C]MSU crystals, a tracer amount of [^14^C]urate was added to the initial urate solution before heating.

#### MSU accumulation assay

To assess accumulation of [^14^C]urate derived from [^14^C]MSU, differentiated THP-1 cells were incubated with [^14^C]MSU (0.2 mg/mL) in RPMI 1640 medium for 2 h at 37 °C. Following the incubation, the medium was removed, and the cells were further cultured in fresh RPMI 1640 for 24 or 48 h. The cells were then lysed, and radioactivity associated with the cell lysate was measured by liquid scintillation counting to determine [^14^C]urate accumulation.

#### ELISA for secreted IL-1β

To evaluate the involvement of ENT3 in MSU-induced inflammatory responses, differentiated THP-1 cells with or without ENT3 knockdown were incubated with MSU crystals (0.2 mg/mL) in RPMI 1640 medium for 2 h at 37 °C. After the incubation, cells were washed and cultured in fresh RPMI 1640 medium for an additional 48 h. Culture supernatants (200 μL aliquots) were collected at each time point. The concentration of IL-1β released into the medium was quantified using an ELISA kit (Proteintech, KE00021) according to the manufacturer’s instructions.

### Quantification and statistical analysis

Kinetic analysis of saturable uptake was performed using the Michaelis–Menten model: *v* = *V*_max_ × *s*/(*K*_m_ + *s*), where *v* represents the uptake rate and *s* is the substrate concentration. The apparent kinetic parameters, *V*_max_ and *K*_m_, were estimated by nonlinear least-squares regression using WinNonlin software (Pharsight, Mountain View, CA), applying 1/*v*^2^ as the weighting factor.

For the estimation of *IC*_50_ values, the profiles of *v* versus inhibitor concentration (*i*) were analyzed using the following equation: *v* = *v*_0_/[1 + (*i*/*IC*_50_)^*n*^]. The *IC*_50_ was estimated similarly by nonlinear least-squares regression, along with the Hill coefficient (*n*), with *v* in the absence of inhibitions (*v*_0_) fixed at the observed value.

Experimental results are expressed as the mean ± SD. Data were obtained from independent biological replicates using separate cell preparations, each assayed in duplicate. Statistical significance was evaluated using Student’s *t* test for two-group comparisons, or one-way or two-way ANOVA followed by Dunnett’s or Bonferroni’s post-hoc test for multiple comparisons. Differences with *p* < 0.05 were considered statistically significant.
