# Supplementary material for: ENT3: A lysosomal urate transporter regulating urate disposition and macrophage inflammation
Source: iScience. 2025 Nov 27;28(12):114249. doi: 10.1016/j.isci.2025.114249 (PMC12752747; doi:10.1016/j.isci.2025.114249)
Supplement: Document S1. Figure S1 and Tables S1–S6 [file mmc1.pdf]

**iScience, Volume *28***

## Supplemental information

# ENT3: A lysosomal urate transporter regulating urate disposition and macrophage inflammation

**Isamu Matake, Tomoya Yasujima, Hirotaka Matsuo, Akiyoshi Nakayama, Yu Toyoda, Tappei Takada, Katsuhisa Inoue, Takahiro Yamashiro, and Hiroaki Yuasa**

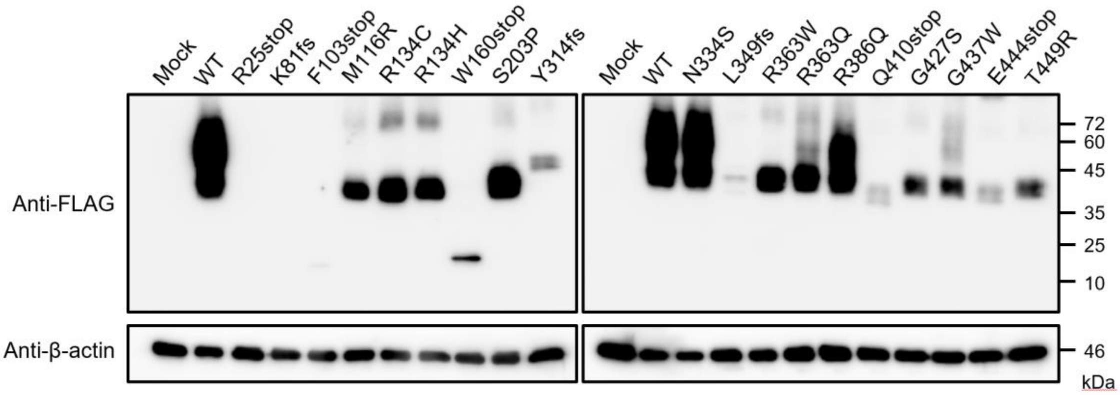


**Supplementary Figure 1.** Protein expression levels of FLAG-ENT3 and its mutants. Western blot analysis was conducted by probing for the FLAG tag, using the whole-cell lysate samples (10 μg protein aliquots) prepared from cells expressing FLAG-tagged ENT3 and its mutants. The blots of β-actin are shown for reference.

**Supplementary Table 1.** Summary of SLC29A3 variants associated with diseases.

| Variants | Frequency | Diseases | References |
|---|---|---|---|
| R25stop | 0.000012* | H syndrome | Noavar et al, 2019 |
| K81fs | unknown | RDD | Noavar et al., 2019 |
| F103stop | 0.0000036** | RDD, SHML | Morgan et al, 2010; Noavar et al., 2019 |
| M116R | 0.000024* | PHID | Cliffe et al, 2009; Morgan et al., 2010; Noavar et al., 2019 |
| R134C | 0.000012* | H syndrome | Priya et al, 2010 |
| R134H | 0.000016* | H syndrome | Al-Haggar et al, 2015 |
| W160stop | 0.000004* | H syndrome | Noavar et al., 2019 |
| S203P | 0.000012* | Dysosteosclerosis | Noavar et al., 2019 |
| Y314fs | unknown | PHID | Cliffe et al., 2009; Morgan et al., 2010; Noavar et al., 2019 |
| N334S | 0.000601* | H syndrome | Noavar et al., 2019 |
| L349fs | unknown | H syndrome | Morgan et al., 2010; Noavar et al., 2019 |
| R363W | 0.000048* | H syndrome | Noavar et al., 2019 |
| R363Q | 0.000008* | H syndrome | Noavar et al., 2019 |
| R386Q | 0.00002* | Dysosteosclerosis | Noavar et al., 2019 |
| Q410stop | 0.000016* | H syndrome | Noavar et al., 2019 |
| G427S | 0.000024* | H syndrome | Al-Haggar et al., 2015; Molho-Pessach et al, 2008; Noavar et al., 2019 |
| G437R | 0.000048* | H syndrome, RDD, PHID | Cliffe et al., 2009; Molho-Pessach et al., 2008; Morgan et al., 2010; Noavar et al., 2019 |
| E444stop | 0.000028* | PHID | Cliffe et al., 2009; Morgan et al., 2010; Noavar et al., 2019 |
| T449R | 0.000007* | PHID | Cliffe et al., 2009; Morgan et al., 2010; Noavar et al., 2019 |

Frequencies were obtained from two datasets in the NCBI database: the gnomAD-Exomes*, comprising 250182 samples, and the gnomADv4-Exomes**, comprising 1401364 samples. fs, frameshift. RDD, Rosai-Dorfman disease; SHML, sinus histiocytosis with massive lymphadenopathy; PHID, pigmented hypertrichotic dermatosis with insulin-dependent diabetes.

**Supplementary Table 2.** Primers for amplification of the cDNA for ENT3.

| PCR | Orientation | Sequence (5' to 3') |
|---|---|---|
| 1st | Forward | GTCCTGGCCGTGCGCCGGAG |
| | Reverse | CCAGGCCAGACATGCTTGATCCCTT |
| 2nd | Forward | GACGAATTCCGACATGGCCGTTGTCTCAG |
| | Reverse | CCAGGCCAGACATGCTTGATCCCTT |

The solid line indicates the restriction site for EcoRI.

**Supplementary Table 3.** Primers for the generation of the cDNA for ENT3-AA.

| Orientation | Sequence (5’ to 3’) |
|---|---|
| Forward | AGGAGGCAGCAGCAGAGAAGCTGCTGGACCGC |
| Reverse | AGCTTCTCTGCTGCTGCCTCCTGGTCAGCTCG |

**Supplementary Table 4.** Primers for the generation of the cDNAs for ENT3-AA mutants.

| Variant | Orientation | Sequence (5' to 3') |
|---|---|---|
| R25stop | Forward | AGTCTCTGAGCTGACCAGGAGGCA |
| (73C>T) | Reverse | CAGCTCAGAGACTGCTGCTTGTGGT |
| K81fs | Forward | GTTCAACTCCGCAACTCCTCCAGC |
| (307, 308del) | Reverse | GCGGAGTTGAACATCCAGTACTCC |
| F103stop | Forward | AACTACTGAGAGCTACCTTGCCGT |
| (347del) | Reverse | CTCTCAGTAGTTCAGGATGTCTGA |
| M116R | Forward | CCTCCAGGCTGTGCCTGGTGGCCAA |
| (347T>G) | Reverse | CACAGCCTGGAGGGCACGGTGGAGG |
| R134C | Forward | CACATCTGTGTCCTGGCCTCACTGA |
| (400C>T) | Reverse | GGACACAGATGTGGACTGCAACCCT |
| R134H | Forward | ACATCCATGTCCTGGCCTCACTGAC |
| (401G>A) | Reverse | AGGACATGGATGTGGACTGCAACCC |
| W160stop | Forward | CCTCCTAGACCCGTGGCTTTTTTGC |
| (479G>A) | Reverse | CGGGTCTAGGAGGAAGTGTCCACCT |
| S203P | Forward | CTGATACCAGGAGGAGCCATGGGCG |
| (607T>C) | Reverse | CTCCTGGTATCAGTGCCTGGGAGTT |
| Y314fs | Forward | GTCACCACGTCTTCTTCATCACCA |
| (940del) | Reverse | AGACGTGGTGACACAGAAGCCCAG |
| N334S | Forward | CCCTCAGCAAGGGTTCGGGCTCACT |
| (1001A>G) | Reverse | CCCTTGCTGAGGGACTCGATGTTGG |
| L349fs | Forward | ATCCCCTCACTACCTTCCTCCTGT |
| (1045del) | Reverse | TAGTGAGGGGATGAAAAACTTGGT |
| R363W | Forward | TGTGGCTGGCAGCTCACCGCCTGGA |
| (1087C>T) | Reverse | GCTGCCAGCCACATAGGTCAGCAAA |
| R363Q | Forward | GTGGCCAGCAGCTCACCGCCTGGAT |
| (1088G>A) | Reverse | AGCTGCTGGCCACATAGGTCAGCAA |
| R386Q | Forward | TCCTCCAGACCTGCCTCATCCCCCT |
| (1157G>A) | Reverse | CAGGTCTGGAGGAGCACGAACCCTG |
| Q410stop | Forward | GTCTTCTAGTCCGATGTGTACCCCG |
| (1228C>T) | Reverse | CGGACTAGAAGACCACAGTCTTCAG |
| G427S | Forward | AGCAACAGCTACCTCAGCACCCTGG |
| (1279G>A) | Reverse | GGTAGCTGTTGCTGAGCCCCAGCAG |
| G437R | Forward | CTCTACAGGCCTAAGATTGTGCCCA |
| (1309G>A) | Reverse | TAGGCCTGTAGAGGAGGGCCAGGGT |
| E444stop | Forward | CCCAGGTAGCTGGCTGAGGCCACGG |
| (1330G>T) | Reverse | CCAGCTACCTGGGCACAATCTTAGG |
| T449R | Forward | AGGCCAGGGGAGTGGTGATGTCCTT |
| (1346C>G) | Reverse | ACTCCCCTGGCCTCAGCCAGCTCCC |

**Supplementary Table 5.** Primers for the real-time PCR analyses of the mRNA expression.

| Protein | Orientation | Sequence (5' to 3') |
|---|---|---|
| ENT3 | Forward | ATGACCGGCTCCTTTCCTATG |
| | Reverse | GCTGTTCCTCACATCACTGGA |
| CD11b | Forward | CCTGTTTCACGGAACCTCAG |
| | Reverse | CAAAATACTGGAGCCTGGGA |
| GAPDH | Forward | CGGAGTCAACGGATTTGGTCGTAT |
| | Reverse | AGCCTTCTCCATGGTGGTGAAGAC |

**Supplementary Table 6.** Sequences of the siRNAs for ENT3.

| Orientation | Sequence (5' to 3') |
|---|---|
| Sense | GCCCUAGAGUUAUUACAAATT |
| Antisense | UUUGUAAUAACUCUAGGGCGA |
